# Influence of cardiorespiratory fitness and body composition on resting and post-exercise indices of vascular health in young adults

**DOI:** 10.1016/j.smhs.2023.11.003

**Published:** 2023-11-21

**Authors:** Rian Q. Landers-Ramos, Kathleen Dondero, Ian Imery, Nicholas Reveille, Hannah A. Zabriskie, Devon A. Dobrosielski

**Affiliations:** a Towson University, Department of Kinesiology, Towson, MD, USA; b University of Maryland School of Medicine, Department of Physical Therapy and Rehabilitation Sciences, Baltimore, MD, USA; c Johns Hopkins University, Department of Cell Biology, Baltimore, MD, USA; d University of Florida, Department of Applied Physiology and Kinesiology, Gainesville, FL, USA

**Keywords:** Cardiorespiratory fitness, Flow mediated dilation, Augmentation index, Vascular, Acute exercise, Body composition

## Abstract

Poor cardiorespiratory fitness may mediate vascular impairments at rest and following an acute bout of exercise in young healthy individuals. This study aimed to compare flow mediated dilation (FMD) and vascular augmentation index (AIx75) between young adults with low, moderate, and high levels of cardiorespiratory fitness before and after an acute bout of aerobic exercise. Forty-three participants (22 men; 21 women) between 18 and 29 years of age completed the study. Participants were classified into low, moderate, and high health-related cardiorespiratory fitness groups according to age- and sex-based relative maximal oxygen consumption ($\dot{V}$O_2_ max) percentile rankings. FMD was performed using Doppler ultrasound and AIx75 was performed using pulse wave analysis at baseline and 60-min after a 30-min bout of treadmill running at 70% $\dot{V}$O_2_ max. A significant interaction (*p* ​= ​0.047; *η*_*p*_^*2*^ ​= ​0.142) was observed, with the moderate fitness group exhibiting a higher FMD post-exercise compared with baseline ([6.7% ​± ​3.1%] vs. [8.5% ​± ​2.8%], *p* ​= ​0.028; *d* ​= ​0.598). We found a significant main effect of group for AIx75 (*p* ​= ​0.023; *η*_*p*_^*2*^ ​= ​0.168), with the high fitness group exhibiting lower AIx75 compared to low fitness group ([−10% ​± ​10%] vs. [2% ​± ​10%], respectively, *p* ​= ​0.019; *g* ​= ​1.07). This was eliminated after covarying for body fat percentage (*p* ​= ​0.489). Our findings suggest that resting FMD and AIx75 responses are not significantly influenced by cardiorespiratory fitness, but FMD recovery responses to exercise may be enhanced in individuals with moderate cardiorespiratory fitness levels.

## Abbreviations

ACSM: American College of Sports Medicine
AEX: aerobic exercise
AIx: augmentation index
AIx75: heart rate-corrected augmentation index
ANCOVA: analysis of covariance
ANOVA: analysis of variance
AP: augmentation pressure
AU: arbitrary unit
AUC: area under the curve
BMI: body mass index
Bpm: beats per minute
*CV*: coefficient of variation
CVD: Cardiovascular disease
*d*: Cohen's *d*
DBP: diastolic blood pressure
dl: deciliter
DXA: dual-energy X-ray absorptiometry
FMD: flow-mediated dilation
*g*: Hedge's *g*
HDL-C: high density lipoprotein cholesterol
HR: heart rate
h: hours
IRB: Institutional Review Board
Kg: kilogram
L: liter
LDL-C: low density lipoprotein cholesterol
L-FMC: low flow-mediated constriction
m: meter
MAP: mean arterial pressure
mg/dL: milligram/deciliter
MHz: megahertz
min: minute
ml: milliliter
mmHg: millimeters of mercury
*η*_*p*_^*2*^: partial eta squared
NO: nitric oxide
PP: pulse pressure
RT: resistance training
SBP: systolic blood pressure
*SD*: standard deviation
s: per second
TG: triglycerides
vs: versus
$\dot{V}$O_2_ max: maximal oxygen consumption
wk: week

## 1 Introduction

Elevated cardiovascular disease (CVD) risk is explained, in part, by impairments in endothelial function (impaired vasodilation),^1^ increased arterial stiffness,^2^^,^^3^ and higher vascular augmentation index (reflective of arterial stiffness and ventricular afterload; AIx75).^4^ Endothelial dysfunction is apparent even in some younger adults^5^ and high AIx75 in young adults is predictive of future CVD risk.^6^ This may be a result of poor cardiorespiratory fitness as low maximal oxygen consumption ($\dot{V}$O_2_max) is an independent risk factor for all-cause and CVD-related mortality in otherwise healthy men and women.^7^ Specifically, low cardiorespiratory fitness is associated with impaired endothelial nitric oxide (NO) production^8^ and inflammation which damages vascular walls and promotes arterial stiffness.^9^ Many vascular outcomes are modifiable through regular exercise^10^^,^^11^ but whether this is related to improvements in cardiorespiratory fitness is debatable.^12^^,^^13^ For example, numerous studies demonstrate improvements in flow-mediated dilation (FMD) in response to exercise training,^14^, ^15^, ^16^ yet FMD in well-trained athletes is often similar to that of age-matched untrained adults due to structural changes in artery diameter.^12^^,^^17^ Further, it is unclear whether high aerobic fitness found in well-trained athletes provides an additive benefit to vascular function over moderate fitness more common in recreational athletes.^18^^,^^19^ This has important public health implications as high levels of cardiorespiratory fitness may not be achievable for many individuals, while moderate fitness may be attainable.

Acute physiological challenges, such as consumption of a high fat meal^20^ or exercise^21^^,^^22^ have been employed to reveal vascular changes that might not otherwise be observed during rest.^23^^,^^24^ Indeed, Dawson et al. proposed that FMD typically experiences a decrease or “nadir” immediately after acute exercise^25^ followed by normalization and/or increase in the later recovery period (30–60 ​min post exercise).^26^ Similarly, AIx75 has been shown to increase immediately following an acute bout of both moderate and high intensity exercise, followed by a gradual return to baseline or improvement within 40–90 ​min into recovery.^27^^,^^28^ However, the time taken for arterial stiffness, a factor influencing AIx75, to recover post-exercise is prolonged in adults with known CVD.^23^^,^^24^ Among other factors hypothesized to influence the vascular recovery response to acute exercise is cardiorespiratory fitness level.^26^ Some studies have compared FMD in elite athletes vs. inactive controls showing a transient decline in FMD in the athletes 1 ​h post-exercise.^17^ However, few have examined FMD or AIx75 recovery responses in healthy young adults based on cardiorespiratory fitness percentiles representative of health-related fitness, rather than elite status.^29^ Investigation into the link between cardiorespiratory fitness and vascular health is warranted, as greater cardiorespiratory fitness achieved when young creates a physiological reserve that can affect vascular responses as we age. Further, an acute bout of exercise can serve as a physiological challenge that reveals how health-related fitness impacts vascular responses to stress. We speculate that examination of indices of vascular health (AIx75 and FMD) in the post-exercise recovery timeframe (∼60 ​min post) may have utility in risk prediction in young healthy adults and that this may be evident in those with low cardiorespiratory fitness.

The purpose of this study was to compare AIx75 and FMD among young adults with low, moderate, and high health-related cardiorespiratory fitness at rest, and 60-min post-exercise. We hypothesized that individuals with low cardiorespiratory fitness would exhibit lower FMD and higher AIx75 at rest compared to those with moderate and high cardiorespiratory fitness. Additionally, we hypothesized that the low fitness group would have an impaired recovery (i.e., lower FMD and higher AIx75 relative to pre-exercise values) 60-min post-exercise compared with the moderate or high fitness groups.

## 2 Materials and methods

### 2.1 Participant recruitment and screening

Participants (*n* ​= ​43; *n* ​= ​22 men and 21 women) were recruited from campus and local advertisements within the Baltimore-Washington, DC region. Participants were healthy, non-smoking, men and women between 18 and 29 years of age. Participants self-reported physical activity and participation in regular cardiorespiratory training (running, cycling, etc.) and resistance training using a brief version of the International Physical Activity questionnaire.^30^ Exclusion criteria for this study was as follows: smokers, men and women previously diagnosed coronary heart disease or congenital heart disease, serum total cholesterol > 200 ​mg/dl, low-density lipoprotein cholesterol (LDL-C) ​> ​130 ​mg/dl, high-density lipoprotein cholesterol (HDL-C) ​< ​35 ​mg/dl, fasting glucose > 100 ​mg/dl, systolic blood pressure (SBP) ​> ​130 ​mmHg, diastolic blood pressure (DBP) ​> ​90 ​mmHg, and body mass index (BMI) ​> ​30 ​kg/m^2^, and women who were currently pregnant. Information about health history, including any medication use, use of hormonal contraceptives, smoking history, and physical activity habits were documented. All women in this study were premenopausal.

### 2.2 Ethical approval

This study was approved by the Towson University Institutional Review Board (IRB #1810041426). Verbal and written informed consent were obtained for all participants and all study procedures adhered to those outlined in the Declaration of Helsinki. All participants provided consent to publish. This study was registed on ClinicalTrials.gov public website (NCT06163456).

### 2.3 Study design

This study consisted of two visits to our laboratory scheduled 48 ​h apart. For each visit, participants were asked to arrive fasted (no food, caffeine, alcohol, medications for ∼12 ​h) and to refrain from exercise for the day prior to their visit. The first visit included anthropometric measures, blood chemistry analyses, and a $\dot{V}$O_2_ max test. Participants were then classified into cardiorespiratory fitness groups based on American College of Sports Medicine (ACSM) criteria for age- and sex-based relative maximal oxygen consumption. Specifically, the low fitness group was classified as $\dot{V}$O_2_ max below the 50^th^ percentile, moderate fitness was classified as between 50^th^-75^th^ percentile, and high fitness was classified as above the 75^th^ percentile.^29^ The second visit included assessment of body composition followed by resting vascular assessments (AIx75 and flow-mediated dilation). Next, participants performed submaximal treadmill exercise for 30 ​min at 70% $\dot{V}$O_2_ max. Vascular assessments were repeated 60 ​min after completion of the exercise. Details of each assessment are described below.

### 2.4 Resting measurements

Seated resting heart rate was assessed from the 10-s radial pulse multiplied by six. Seating resting blood pressure was measured with a standard sphygmomanometer on the brachial artery of the participant's dominant arm. Height and weight were measured, and BMI was calculated as body weight in kg/body height in m^2^. A small blood sample was collected to confirm that all participants were free from traditional cardiometabolic risk factors. Blood was collected via finger prick and placed in a CardioChek® analyzer (PTS Diagnostics, Whitestown, IN) to assess total cholesterol, HDL-C, LDL-C, triglycerides (TG), and glucose levels.

### 2.5 Maximal oxygen consumption

$\dot{V}$O_2_ max was assessed during the first visit using a constant-speed treadmill protocol with a 2% increase in incline every 2 ​min until exhaustion. Participants warmed up for 5–10 ​m jogging at a self-selected speed. The treadmill speed was chosen based on each participant's experience, typical running speed, and heart rate such that $\dot{V}$O_2_ max was achieved in ∼6–12 ​min. Pulmonary ventilation and expired gas concentrations were analyzed in real time using an automated computerized indirect calorimetry system (Parvo Medics TrueOne 2400, Salt Lake City, UT) and data were averaged every 30 ​s. $\dot{V}$O_2_ was considered maximum if a plateau was achieved (increase in $\dot{V}$O_2_ of < 150 ​ml/min with increased work) or, in the absence of a clear plateau, tests were verified to meet at least two of the following secondary criteria of maximal effort: a respiratory exchange ratio > 1.10, a rating of perceived exertion > 18 on a 6–20 scale, and a heart rate within 10 beats/min of the age-predicted maximum.^29^^,^^31^ Heart rate was measured during the test using chest strap heart rate monitors (Polar Electro Inc, Lake Success, NY). At the end of the test, participants walked for 5–10 ​min to cool down.

### 2.6 DXA

We previously found body fat percentage to be a meaningful covariate of AIx75.^22^ Thus, body composition was assessed to better characterize fitness groups, and so that body fat percentage may be considered as a covariate in our analysis. Body composition of all participants was measured by dual-energy X-ray absorptiometry (DXA) scan (Lunar Prodigy X-Ray Bone Densitometer, GE Healthcare, Chicago, IL) per methods previously described.^32^^,^^33^ Briefly, participants were positioned on the DXA table by trained members of the research team. Quality assurance measures were performed daily (average CV over the study period 3.19%). All scans were analyzed using enCORE software (version 14.0) according to the manufacturer. Percent body fat was recorded for all participants.

### 2.7 Pulse wave analyses and central blood pressures

Participants rested in the supine position for 10-min before central blood pressures were measured with an automated device using specialized cuff-based brachial waveform analysis (SphygmoCor, AtCor Medical Sydney, NSW, Australia). Aortic pressure waveforms were reconstructed from the brachial artery pressure waveforms using a generalized validated transfer function.^34^ AIx was defined as the differences between the first and second systolic peak divided by pulse pressure. AIx75 is expressed as a percentage and corrected for a heart rate of 75 bpm.^35^ Other computed measures were included to aid in interpretation of pulse wave analyses findings and included aortic systolic and diastolic blood pressure (SBP and DBP, respectively), aortic pulse pressure (PP), augmentation pressure (AP), and mean arterial pressure (MAP).

### 2.8 Flow-mediated dilation

Endothelium-dependent vasodilation was evaluated via FMD of the brachial artery on the dominant arm at baseline, and 60-min after completion of the acute exercise bout as per published guidelines.^36^ This technique utilizes Doppler ultrasound of the brachial artery to assess the nitric oxide-mediated vasodilatory response to increased shear stress following a 5-min arterial occlusion. Assessments were performed by a single experienced sonographer using previously published methodology.^37^^,^^38^ Brachial artery diameter and pulse-wave Doppler velocity signals were acquired simultaneously using a high-resolution ultrasound (GE Logiq, GE Healthcare Products, USA) equipped with a 7.5–12 ​MHz linear array transducer. For all FMD measurements, the participants rested in a supine position for 10 ​min. Then the ultrasound probe was placed parallel to the brachial artery and stabilized with a probe holder keeping the probe at an insonation angle of < 60°–70°. To minimize within-subject variability, a marker was used to outline the placement of the probe along the participant's arm and kept consistent for each test. Reactive hyperemia was induced with the following protocol: A pneumatic cuff (Hokanson, Bellevue, WA) was placed around the thickest region of the forearm, distal to the ultrasound measurement site. Baseline arterial diameter and blood velocities were recorded for 2 ​min. The cuff was then inflated to a suprasystolic pressure (> 220 ​mmHg) for 5 ​min to induce forearm ischemia keeping the pressure consistent between participants and tests. The cuff was then released, and arterial diameter and blood velocity were recorded continuously for 3 ​min to measure endothelial response to reactive hyperemia. Brachial artery diameters and blood velocity were recorded continuously using video capture (El Gato; San Fransicso, CA). Analyses of all files were conducted offline using automated edge-detecting software (QUIpU Cardiovascular Suite, FMD Studio; Pisa, Italy) with the operator blinded to participants and condition. Resting diameter represented the average 2-min baseline recording and maximal diameter was considered the largest diameter recorded following cuff deflation determined using a 5-s time bin to account for outliers. FMD was calculated as the percentage change in arterial diameter from baseline to the maximal per current FMD guidelines^38^^,^^39^ as follows: [(Maximal diameter-Baseline diameter)/Baseline diameter] × 100. Shear rate (s^−1^) was quantified using Doppler and reported as baseline, peak, and area under the curve (AUC). The AUC was calculated by summing the areas of successive post-occlusion trapezoids from baseline until peak dilation of the brachial artery. FMD/AUC has been found to distinguish differences between groups based on cardiovascular risk^40^ which was deemed appropriate based on our independent variable, $\dot{V}$O_2_ max percentile, being a major cardiovascular risk factor in otherwise healthy adults.^7^ To allow for a comprehensive analysis, FMD is presented as %FMD and was also assessed normalized to brachial artery diameter and Shear AUC.

### 2.9 Submaximal exercise

After a 5–10-min warmup at a self-selected speed, participants performed 30 ​min of walking or running on a treadmill at 70% of their maximal oxygen consumption. Participants were first fitted with a mask and oxygen consumption was recorded using the metabolic cart as described above. As described previously,^41^^,^^42^ the speed and/or incline of the treadmill was adjusted until participants achieved 70% their $\dot{V}$O_2_ max based on the results from the test performed during the initial visit.^29^ This intensity was selected as it provides the recommended stimulus for most cardiovascular adaptations.^19^^,^^43^ After reaching steady state (∼5–10 ​min), the mask was removed while participants continued their exercise. Intensity was maintained based on the corresponding heart rate reserve calculated from the resting heart rate and max heart rate achieved during the $\dot{V}$O_2_ max test.

### 2.10 Statistical analysis

Sample size calculations were performed to determine the number of subjects in each fitness category needed to determine significant differences in the major outcomes of interest (i.e., FMD and AIx75) at rest and following acute exercise. Calculations were based on effect size estimates from findings published in the literature regarding our primary study outcomes: %FMD,^17^^,^^44^^,^^45^ and AIx75^21^^,^^27^^,^^46^ at rest and in response to acute exercise. These calculations revealed a sample size of 12 per group needed to yield 80%–95% power.^47^ All data statistical analyses were performed using SPSS v.25 (SPSS, Inc. Chicago, IL). Assumptions of normality and sphericity were met for all outcome measures. One-way analysis of variance (ANOVA) was used to determine differences in subject characteristics across groups. Pearson correlation coefficients were performed to determine whether there was a relationship between cardiorespiratory fitness and FMD or AIx75 at rest and post-exercise. A 2 (time) × 3 (group) repeated measured ANOVA was used to determine whether differences in major outcome variables were present across cardiorespiratory fitness groups before or after acute exercise. We have previously reported that body fat percentage is a significant predictor of AIx75 in young healthy adults,^22^ so AIx75 was also analyzed using a repeated measures analysis of covariance (ANCOVA) with body fat percentage as a covariate. When an interaction or main effect was noted, post-hoc pairwise comparisons were performed using Bonferroni corrections. The α level was set *a priori* for all statistical procedures at *α* ​= ​0.05. Effect sizes were calculated for all statistically significant comparisons. Effect sizes for 2-way ANOVAs are presented as partial eta-squared (*η*_*p*_^*2*^). The effect was determined trivial if ​< ​0.01, small if the effect size was between 0.01 and 0.06; medium if between 0.06 and 0.14, large if ​> ​0.14.^48^ For effect size calculations between different time points within the same group Cohen's *d* was used. For effect size calculations between groups, Hedge's *g* was calculated. For these results, the effect was determined trivial if ​< ​0.2, small if the effect size was between 0.2 and 0.5; medium if between 0.5 and 0.8, large if ​> ​0.8.^49^ Statistical significance was accepted at a *p*-value of ≤ 0.05. Data are presented as means ​± ​*SD*.

## 3 Results

### 3.1 Participant characteristics

A total of 43 individuals took part in the study (*n* ​= ​22 men and 21 women). Participant characteristics are found in Table 1. A total of *n* ​= ​2 individuals did not exhibit a clear plateau in $\dot{V}$O_2_ but both participants exhibited other criteria outlined in methods for maximal effort^31^ and so were still included in the study. As expected, $\dot{V}$O_2_ max and average minutes of aerobic exercise performed per week was significantly higher in the moderate and high fitness groups compared with the low fitness group (*p* ​< ​0.000 1 for both). The high fitness group also performed nearly twice as much aerobic exercise per week compared with the moderate fitness group (*p* ​< ​0.009). The moderate and high fitness groups exhibited lower BMI (*p* ​= ​0.01 and *p* ​< ​0.000 1, respectively) and body fat percentage compared with the low fitness group (*p* ​= ​0.008 and *p* ​< ​0.000 1, respectively). Further, the high fitness group exhibited significantly lower BMI compared with the moderate fitness group (*p* ​= ​0.024). Of the 21 women who participated in the study, hormonal contraceptives were used by 10 women (3 low fitness, 1 moderate fitness, 6 high fitness), one of whom reported use of an implantable device and absence of withdrawal bleeding. The other 11 women reported regular menstrual cycles with 5 in the follicular phase (1 moderate fitness, 4 high fitness) and 6 in the luteal phase (2 low fitness, 3 moderate fitness, 1 high fitness) at the time of testing.

**Table 1:** Participant characteristics.

Variables	Low Fitness (*n* ​= ​13)	Moderate Fitness (*n* ​= ​14)	High Fitness (*n* ​= ​16)
Sex (M/W)	8/5	9/5	5/11
Age (yrs)	22 ​± ​3	22 ​± ​2	23 ​± ​3
SBP (mmHg)	113 ​± ​10	113 ​± ​10	110 ​± ​10
DBP (mmHg)	72 ​± ​8	71 ​± ​8	68 ​± ​8
Height (m)	1.71 ​± ​0.09	1.70 ​± ​0.11	1.68 ​± ​0.97
Weight (kg)	80.5 ​± ​15.1	71.7 ​± ​11.8	62.1 ​± ​12.5∗
BMI (kg/m^2^)	27 ​± ​4	24 ​± ​2∗	21 ​± ​3∗#
Body Fat (%)	32 ​± ​7	25 ​± ​7∗	22 ​± ​6∗
Total Cholesterol (mg/dL)	125 ​± ​27	139 ​± ​33	134 ​± ​24
HDL-C (mg/dL)	47 ​± ​10	56 ​± ​15	65 ​± ​18∗
LDL-C (mg/dL)	80 ​± ​20	66 ​± ​22	60 ​± ​30
Triglycerides (mg/dL)	66 ​± ​24	88 ​± ​47	67 ​± ​17
Glucose (mg/dL)	91 ​± ​11	82 ​± ​13	82 ​± ​12
Maximal oxygen consumption (L/min)	2.9 ​± ​1.1	3.6 ​± ​0.9	3.3 ​± ​0.9
Maximal oxygen consumption (mL/kg/min)	36.6 ​± ​10.4	49.5 ​± ​6.2∗	54.1 ​± ​7.4∗
Average AEX (min/wk)	33 ​± ​63	130 ​± ​140∗	250 ​± ​135∗#
Average RT (min/wk)	78 ​± ​159	167 ​± ​221	121 ​± ​136

∗denotes statistically different than low fitness; #denotes statistically different than moderate fitness. Data are presented as means ​± ​*SD*.

M/W, men/women; yrs, years; SBP, systolic blood pressure; DBP, diastolic blood pressure; mmHg, millimeters of mercury; m, meter, kg, kilogram; BMI, body mass index; mg/dL, milligram per deciliter; HDL-C, high density lipoprotein cholesterol; LDL-C, low-density lipoprotein cholesterol; L/min, liters per minute; mL/kg/min, mililiters per kilogram of body weight per minute; AEX, aerobic exercise; RT, resistance training; min/wk, minutes per week.

### 3.2 Correlates of cardiorespiratory fitness, flow mediated dilation, and augmentation index

There were no significant correlations between relative $\dot{V}$O_2_ max and FMD at rest (*r* ​= ​0.192; *p* ​= ​0.217) or post-exercise (*r* ​= ​−0.069; *p* ​= ​0.661). Both resting AIx75 (*r* ​= ​−0.488; *p* ​= ​0.001) and post-exercise AIx75 (*r* ​= ​−0.311; *p* ​= ​0.037) were negatively correlated with relative $\dot{V}$O_2_ max.

### 3.3 Brachial artery diameter, shear rate, and flow-mediated dilation

Resting and post-exercise values for brachial artery diameter, shear rate, and FMD can be found in Table 2. We found no group∗time interaction (*p* ​= ​0.880; *η*_*p*_^*2*^ ​= ​0.006), main effect of time (*p* ​= ​0.619; *η*_*p*_^*2*^ ​= ​0.006), or group (*p* ​= ​0.228; *η*_*p*_^*2*^ ​= ​0.071) on resting brachial artery diameter. Similarly, there was no group∗time interaction (*p* ​= ​0.450; *η*_*p*_^*2*^ ​= ​0.039), main effect of time (*p* ​= ​0.435; *η*_*p*_^*2*^ ​= ​0.015), or main effect of group (*p* ​= ​0.110; *η*_*p*_^*2*^ ​= ​0.105) on peak brachial artery diameter. Analyses of shear rate AUC revealed no time∗group interaction (*p* ​= ​0.643; *η*_*p*_^*2*^ ​= ​0.022), nor main effects of time (*p* ​= ​0.439; *η*_*p*_^*2*^ ​= ​0.015) or group (*p* ​= ​0.117; *η*_*p*_^*2*^ ​= ​0.104). There was a significant group∗time interaction for %FMD (*p* ​= ​0.047; *η*_*p*_^*2*^ ​= ​0.142) with the moderate fitness group exhibiting significantly higher %FMD post-exercise vs. baseline (*p* ​= ​0.028; *g* ​= ​0.598). There was no main effect of time (*p* ​= ​0.248; *η*_*p*_^*2*^ ​= ​0.033) or group (*p* ​= ​0.128; *η*_*p*_^*2*^ ​= ​0.098). When normalized to shear rate (FMD/Shear rate AUC), there was no group∗time interaction (*p* ​= ​0.920; *η*_*p*_^*2*^ ​= ​0.004) or main effect of time (*p* ​= ​0.130; *η*_*p*_^*2*^ ​= ​0.059), but we found a main effect of group (*p* ​= ​0.041; *η*_*p*_^*2*^ ​= ​0.155). Regardless of timepoint, the moderate fitness group exhibited significantly higher FMD/Shear rate AUC compared with the low fitness group (*p* ​= ​0.037; *g* ​= ​1.02). The high fitness group was not significantly different than the moderate (*p* ​= ​0.870*;* *g* ​= ​0.399) or low fit group (*p* ​= ​0.369; *g* ​= ​0.621). FMD was also analyzed relative to brachial artery diameter (FMD/baseline diameter). There was a significant group∗time interaction (*p* ​= ​0.041; *η*_p_^*2*^ ​= ​0.148). Specifically, the moderate fitness group exhibited significantly higher FMD/baseline diameter post-exercise (*p* ​= ​0.034; *d* ​= ​0.504). We observed no main effects of time (*p* ​= ​0.195; *η*_p_^*2*^ ​= ​0.042) or group (*p* ​= ​0.483; *η*_p_^*2*^ ​= ​0.036).

**Table 2:** Flow-mediated dilation at rest and post-exercise.

Variables	Time	Group			*p*-value		
		Low fitness	Moderate fitness	High fitness	Group	Time	Group∗Time Interaction
**Baseline Diameter (mm)**	*Resting*	3.6 ​± ​0.5	3.9 ​± ​0.5	3.6 ​± ​0.6	0.228	0.619	0.880
	*Post-Exercise*	3.6 ​± ​0.6	3.9 ​± ​0.5	3.6 ​± ​0.7			
**Peak Diameter (mm)**	*Resting*	3.7 ​± ​0.5	4.1 ​± ​0.5	3.9 ​± ​0.6	0.110	0.435	0.450
	*Post-Exercise*	3.8 ​± ​0.6	3.8 ​± ​0.8	3.9 ​± ​0.7			
**Shear Rate AUC (AU)**	*Resting*	9.5 ​× ​10^4^ ​± ​1.4 ​× ​10^4^	2.5 ​× ​10^4^ ​± ​1.1 ​× ​10^4^	3.5 ​× ​10^4^ ​± ​2.7 ​× ​10^4^	0.117	0.439	0.643
	*Post-Exercise*	9.3 ​× ​10^4^ ​± ​1.6 ​× ​10^4^	6.9 ​× ​10^4^ ​± ​1.1 ​× ​10^4^	4.2 ​× ​10^4^ ​± ​8.0 ​× ​10^4^			
**FMD (%)**	*Resting*	4.9 ​± ​2.5	6.7 ​± ​3.1	6.7 ​± ​3.8	0.128	0.248	**0.047**
	*Post-Exercise*	5.9 ​± ​3.3	8.5 ​± ​2.8#	5.7 ​± ​3.4			
**FMD/Shear AUC (AU)**	*Resting*	0.1^−3^ ​± ​0.07^−3^	0.3^−3^ ​± ​0.27^−3^∗	0.2^−3^ ​± ​0.11^−3^	**0.041**	0.130	0.920
	*Post-Exercise*	0.2^−3^ ​± ​0.14^−3^	0.4^−3^ ​± ​0.36^−3^∗	0.4^−3^ ​± ​0.49^−3^			
**FMD/diameter (AU)**	*Resting*	1.5 ​± ​0.8	1.8 ​± ​0.9	1.9 ​± ​1.0	0.483	0.195	**0.041**
	*Post-Exercise*	1.8 ​± ​1.2	2.2 ​± ​0.9#	1.7 ​± ​0.9			

∗Indicates significantly different than low fitness group. #Indicates significant within group difference from rest. Data are presented as means ± *SD*. mm, milimeters; AUC, area under the curve; FMD, flow-mediated dilation; AU, arbitrary units.

### 3.4 Central blood pressures and augmentation index

Resting and post-exercise values for central blood pressures and augmentation index results can be found in Table 3. No significant interactions were found for any blood pressure outcomes (*p* -values between 0.643 and 1.00). There was a significant main effect of time for aortic SBP (*p* ​= ​0.001; *η*_*p*_^*2*^ ​= ​0.245), aortic pulse pressure (*p* ​= ​0.001; *η*_*p*_^*2*^ ​= ​0.236), and HR (*p* ​< ​0.001; *η*_*p*_^*2*^ ​= ​0.412), but not for aortic DBP (*p* ​= ​0.957), AP (*p* ​= ​0.330), or MAP (*p* ​= ​0.311). SBP and PP were lower post-exercise (*p* ​= ​0.001 for both), and HR was higher post-exercise (*p* ​< ​0.001). There was no main effect for group on aortic SBP (*p* ​= ​0.550), DBP (*p* ​= ​0.102), PP (*p* ​= ​0.565), AP (*p* ​= ​0.383), or MAP (*p* ​= ​0.065). There was a significant main effect of group on HR (*p* ​< ​0.002; *η*_*p*_^*2*^ ​= ​0.257) with low fitness exhibiting higher HR than both the moderate and high fitness group (*p* ​= ​0.021; *g* ​= ​1.06 and *p* ​= ​0.002; *g* ​= ​1.38, respectively).

**Table 3:** Central blood pressures (mmHg) and augmentation index.

Variables	Time	Group			*p*-value		
		Low Fitness	Moderate Fitness	High Fitness	Time	Group	Group∗Time Interaction
**Aortic SBP**	*Resting*	115 ​± ​15	112 ​± ​8	113 ​± ​9	**0.001**	0.550	1.00
	*Post-Exercise*	111 ​± ​8	107 ​± ​8	108 ​± ​7			
**Aortic DBP**	*Resting*	84 ​± ​9	78 ​± ​10	80 ​± ​7	0.957	0.102	0.666
	*Post-Exercise*	83 ​± ​7	78 ​± ​6	81 ​± ​7			
**Aortic PP**	*Resting*	31 ​± ​8	34 ​± ​10	33 ​± ​8	**0.001**	0.565	0.699
	*Post-Exercise*	28 ​± ​4	29 ​± ​8	27 ​± ​5			
**AP**	*Resting*	1 ​± ​5	−0.4 ​± ​5	−1 ± 5	0.330	0.383	0.643
	*Post-Exercise*	−0.4 ​± ​4	−2 ± 4	−0.9 ​± ​4			
**MAP**	*Resting*	98 ​± ​11	91 ​± ​8	92 ​± ​8	0.311	0.065	0.797
	*Post-Exercise*	96 ​± ​8	90 ​± ​6	92 ​± ​7			
**HR**	*Resting*	73 ​± ​8	62 ​± ​8∗	60 ​± ​12∗	**< 0.001**	**0.002**	0.787
	*Post-Exercise*	83 ​± ​13	73 ​± ​16∗	68 ​± ​9∗			
**AIx (%)**	*Resting*	2.5 ​± ​14.3	−1.3 ​± ​15.4	−5.4 ​± ​15.6	Unadjusted *p*-values		
					0.168	0.359	0.905
	*Post-Exercise*	−1.8 ​± ​14.4	−3.3 ​± ​17.6	−8 ​± ​13.7	Adjusted *p*-values^a^		
					0.317	0.611	0.876
**AIx75 (%)**	*Resting*	1.7 ​± ​12.7	−5.1 ​± ​10.1	−10.1 ​± ​14.7∗	Unadjusted *p*-values		
					0.673	**0.023**	0.893
	*Post-Exercise*	1.8 ​± ​14.8	−2.8 ​± ​11.8	−9.8 ​± ​13.1∗	Adjusted *p*-values^a^		
					0.064	0.489	0.60

Data are presented as means ​± ​*SD*.

∗denotes statistically different than low fitness across time.

^a^Adjusted *p*-values were obtained after covarying for body fat percentage.

SBP, systolic blood pressure; DBP, diastolic blood pressure; PP, pulse pressure; AP, augmentation pressure; MAP, mean arterial pressure; HR, heart rate; AIx, augmentation index; AIx75, heart rate-corrected augmentation index.

Body fat percentage was a significant predictor of resting AIx (*r* ​= ​0.541; *p* ​< ​0.001) and resting AIx75 (*r* ​= ​0.610; *p* ​< ​0.001). There was no group∗time interaction (*p* ​= ​0.905; *η*_*p*_^*2*^ ​= ​0.005), main effect of time (*p* ​= ​0.168; *η*_*p*_^*2*^ ​= ​0.044) or main effect of group (*p* ​= ​0.359; *η*_*p*_^*2*^ ​= ​0.046) for AIx. These results remained non-significant when adjusting for body fat percentage. When normalized to a heart rate of 75 bpm, the time∗group interaction (*p* ​= ​0.893; *η*_*p*_^*2*^ ​= ​0.005) and main effect of time (*p* ​= ​0.673; *η*_*p*_^*2*^ ​= ​0.004) were still absent. We noted a significant main effect of group for AIx75 (*p* ​= ​0.023; *η*_*p*_^*2*^ ​= ​0.168). The high fitness group had significantly lower AIx75 compared to low fitness group (*p* ​= ​0.019; *g* ​= ​1.07) but there were no differences in AIx75 between the high fitness and moderate fitness group (*p* ​= ​0.407). However, when body fat percentage was added as a covariate, the significant difference between high and low fitness groups was eliminated (*p* ​= ​0.489; *η*_*p*_^*2*^ ​= ​0.035).

## 4 Discussion

The benefits of regular exercise training on vascular health and the associated reduction of CVD risk are well understood and may be met through various modes of exercise.^1^^,^^11^ Whether higher cardiorespiratory fitness level influences the resting or acute vascular responses to exercise in younger adults is less understood. Our study extends the literature in several novel ways. First, we confirm that health-related cardiorespiratory fitness does not affect resting FMD in young, healthy individuals and demonstrate that individuals with moderate cardiorespiratory fitness may exhibit more favorable FMD responses in the 60-min post exercise recovery period compared to those with low or high cardiorespiratory fitness. Further, we report that after controlling for body fat percentage, AIx75 values are not different between low fit vs. high fit individuals. Finally, we revealed that health-related cardiorespiratory fitness does not influence post-exercise AIx75 responses. Our findings suggest that although low cardiorespiratory fitness is a major risk factor for CVD, risk may not be explained by these vascular measures at rest or in response to exercise among young healthy adults.

In the present study, $\dot{V}$O_2_ max was not correlated with FMD, and we did not find any differences in resting FMD across fitness groups. Several studies have previously documented that regular exercise training can improve FMD^14^^,^^16^^,^^50^^,^^51^ and this has been modestly associated with improvements in $\dot{V}$O_2_ max.^16^ However, conflicting evidence has been reported with some showing that well-trained athletes do not exhibit greater FMD than inactive controls.^12^^,^^13^ In line with our findings, Bell et al. found that there were no differences in FMD between young men of high and low fitness categories, suggesting that cardiorespiratory fitness levels may not influence endothelial vasoreactivity responses in young healthy individuals.^45^ A recent meta-analysis by Montero et al. found that masters athletes, but not young athletes, exhibit better FMD compared with age-matched sedentary controls^13^ suggesting that the relationship between aerobic fitness and resting FMD is age dependent. Many studies that did not report differences in FMD according to fitness level found evidence of vascular remodeling in highly trained athletes in the form of greater brachial artery diameter.^12^^,^^17^^,^^18^ However, we did not observe differences between groups in brachial artery diameter. Our groups were determined by ACSM percentiles for health-related cardiorespiratory fitness,^29^ whereas other studies selected highly trained and nationally or internationally ranked endurance athletes.^17^^,^^18^ Thus, changes in vascular remodeling may be more evident in elite-level athletes. Our study did have a higher proportion of women in the high fitness group. To account for differences in body size that may have impacted our results, we also analyzed FMD relative to brachial artery diameter and this did not change our results. However, we cannot exclude the possibility that the unequal proportion of men and women at least partly explains why our results are not consistent with some previous work.

It has been suggested that FMD exhibits a “biphasic” response to acute exercise.^26^ Specifically, FMD has been found to decrease immediately after exercise^25^ followed by normalization and/or improvement in the later recovery period ∼30–60 ​min post-exercise.^26^ We found that only the moderate fitness group exhibited greater FMD in the recovery period following acute submaximal aerobic exercise. Increases in FMD have been observed previously in recreationally active individuals 60-min post-exercise.^52^^,^^53^ Additionally, Kapilevich et al. previously noted that FMD increased immediately post-exercise in moderately trained, but not well-trained, individuals, potentially due to disturbances in redox balance and subsequent reductions in NO bioavailability in the highly trained individuals.^44^ Indeed, Goto et al. found that exercise training at moderate-intensities, but not mild- or high-intensities improved endothelium-dependent vasodilation due to greater NO production and lower oxidative stress.^19^ Mechanistically, the application of these findings to an acute bout of exercise would suggest that moderately trained individuals exhibit greater oxidative balance which would allow them to respond more favorably to a single exercise stressor. While not significantly different between groups, the moderate-fitness group reported numerically greater time spent performing resistance training.^19^ Traditionally, vascular improvements are associated with aerobic exercise.^16^ However, a recent meta-analysis concluded that resistance training also results in clinically relevant improvements in endothelial function.^54^ It is possible that the combined influence of resistance and aerobic exercise enhances resilience to an acute exercise challenge through a greater oxidative balance. Shear rate has been found to affect the FMD response.^40^ We did not find differences in shear rate, and when FMD was analyzed relative to shear rate, we no longer saw a significant interaction. A group effect remained with the moderately fit group exhibiting higher FMD at both timepoints. These results suggest that while moderately fit young adults still exhibit more optimal FMD responses, the “biphasic” response in this group post-exercise is not evident when shear rate is similar.

We found a significant negative association between relative $\dot{V}$O_2_ max and AIx75 which is in line with other studies.^55^^,^^56^ Additionally, we found that regardless of timepoint, AIx75 was lower in individuals with higher cardiorespiratory fitness compared to those with low cardiorespiratory fitness. While the exact mechanisms behind this cannot be confirmed given the nature of our study, anti-inflammatory effects of regular exercise and decreased sympathetic tone may explain our findings.^56^ However, group differences were eliminated when body fat percentage was added as a covariate. We have previously found that adjusting for body fat percentage eliminated differences in resting AIx75 between men and women.^22^ Interestingly, in both the present study and our previous study, all individuals had body fat percentages within healthy ranges.^57^ Body fat percentage has been previously found to correlate with other indices of vascular stiffness^58^^,^^59^ suggesting that even small differences in body fat may influence AIx75 in healthy young adults. Greater adiposity is often accompanied by inflammation and oxidative stress which can reduce artery compliance.^58^ Indeed, we found that for a single percent increase in body fat, AIx75 increased 1.1%. A 10% absolute increase in AIx is associated with an age-and risk-factor -adjusted pooled relative risk of 0.138 4 for all-cause mortality.^4^ This suggests that although the body fat percentage and AIx75 values for individuals in the low fitness group are still in the normal range,^6^ the differences are physiologically meaningful especially when paired with lower cardiorespiratory fitness. As with our other findings, our results may be influenced by the higher prevalence of women in the high fitness group. However, as women typically have more body fat than men, the differences that we observed prior to controlling for body fat are more likely explained by the lower body fat found in endurance athletes.

We did not detect any differences in AIx75 either between or within groups in the post-exercise time point. We observed a post-exercise reduction in SBP that is consistent with previous reports.^60^^,^^61^ This contributes to the post-exercise reduction in PP suggesting that pulse wave reflection was also reduced consistently across groups post-exercise. Our findings are in line with some,^21^^,^^46^ but not all^27^ previous studies looking at AIx75 recovery responses 60-min following moderate continuous exercise.^21^^,^^27^^,^^46^ Perissiou et al. compared AIx75 responses to moderate continuous exercise in older men and women ([72 ​± ​5] yrs old) in low-, mid-, and high-fitness categories.^27^ Compared to the group with low cardiorespiratory fitness, AIx75 was ∼9% lower at rest in the high fit group. However, AIx75 was significantly lower 60-min post exercise across all groups^27^ suggesting that age, and not cardiorespiratory fitness, impacts AIx75 responses to acute exercise. Currently, the clinical relevance of AIx75 recovery responses to exercise remain to be determined, especially in younger adults, but does not seem to be affected by cardiorespiratory fitness.

This study did have some limitations. We chose to assess the effects of a submaximal aerobic exercise bout due to vascular adaptations occurring largely in response to aerobic exercise,^10^^,^^16^ and moderate continuous exercise eliciting the most favorable vascular responses.^19^ However, a more significant stressor may be needed to reveal differences across fitness levels in young healthy individuals. It is also possible that our method for assessing intensity (initial $\dot{V}$O_2_ followed by associated heart rate reserve) may have resulted in slight individual variability during the submaximal test. Additionally, some studies have found that FMD and AIx75 exhibit different responses^17^^,^^21^^,^^27^^,^^46^ or no significant changes^44^^,^^62^ in response to acute exercise. Future studies should assess at multiple time points to determine whether differences across fitness categories might be observed sooner after completion of exercise which may be predictive of greater future cardiovascular risk. AIx75 provides a composite measure of arterial stiffness and cardiac afterload,^63^ whereas FMD assesses vascular reactivity. Considering the discrepant findings between FMD and AIx75 in the present study, the mechanisms through which cardiorespiratory fitness may affect vascular health seem to vary. Future studies should consider additional vascular measures, including pulse wave velocity as the gold standard assessment unique to arterial stiffness, to provide a more comprehensive assessment of vascular health across those with different cardiorespiratory fitness levels. Finally, despite being powered for most comparisons, sample size estimates indicated that we may not be adequately powered to detect differences between the low- and moderate-fitness groups for AIx75 when including a covariate. We opted to include results from this analysis both with and without body fat as a covariate as we were powered to detect differences between our other groups, and previous work in our lab has indicated body fat as a significant covariate when considering AIx75.^22^ Future studies may need to be performed with a greater number of participants to confirm our findings, particularly for AIx75 when covariates are employed.

These limitations are balanced with the following strengths. In addition to studying young adults on the upper and lower ends of the spectrum of cardiorespiratory fitness, we chose to include a moderate fitness group. As a large percentage of young adults in the United States do not meet the minimum physical activity guidelines,^64^ it is important to include this group that represents a fitness level that is attainable for most adults (50^th^ percentile). In the present study, the moderate fitness group not only exhibited the most favorable post-exercise response to FMD but also had AIx75 values similar to the higher fitness group. Further, they exhibited optimal values in fat free mass and body fat percentage. Thus, from a public health perspective, our findings can be used to promote physical activity recommendations for vascular health that are achievable for the larger population. While this study was performed in younger adults our findings have implications for older populations as well. Reductions in $\dot{V}$O_2_ max,^65^ increases in body fat percentage, and reductions in muscle mass^66^ occur with advancing age. Our study suggests that the degree to which body composition changes take place in relation to declines in cardiorespiratory fitness may affect the progression of some vascular impairments. Based on our findings, regularly incorporating both aerobic and strength training may help to best to improve body composition and preserve vascular health, while offsetting declines in cardiorespiratory fitness. Another strength of our study was the inclusion of both men and women. We documented, but did not control for, menstrual cycle in this study. Whether menstrual cycle should be controlled for in vascular studies has been recently debated.^67^^,^^68^ As our study was not designed to mechanistically determine the effects of estrogen on the vascular recovery responses to exercise, nor were we seeking to compare results between sexes, controlling for menstrual cycle was not deemed necessary in this study. However, we cannot discount that the menstrual cycle phase and/or hormonal contraceptive use may be playing some role in the vascular responses to exercise. Specifically, some studies have found that endothelial function is higher during the late follicular phase of the menstrual cycle,^69^^,^^70^ compared with the early follicular phase,^71^ whereas multiple studies have demonstrated that vascular function is stable across the menstrual cycle and oral hormonal contraceptive cycle.^70^^,^^72^, ^73^, ^74^ While it is possible that the greater proportion of women in the high-fitness group may have impacted our results, all but one of the women in that group were either in the early follicular phase or were taking hormonal contraceptives. Thus, if there was an effect of the menstrual cycle or use of oral contraceptives on our study outcomes, it was likely minimal.

## 5 Conclusions

In conclusion, we found that cardiorespiratory fitness levels do not influence resting FMD, but moderately trained young adults may exhibit more favorable recovery responses to an acute bout of aerobic exercise. Additionally, we found that lower AIx75 in high aerobically fit adults was no longer present when controlling for body fat percentage. Collectively, these findings indicate that amongst young healthy adults, resting FMD and AIx75 responses are not significantly influenced by cardiorespiratory fitness, but FMD recovery responses to exercise may be enhanced in individuals with moderate cardiorespiratory fitness levels.

## Submission statement

All authors have read and agree with the manuscript content. While this manuscript is being reviewed for the Sports Medicine and Health Science it will not be submitted elsewhere for review and publication.

## Ethical approval statement

This study was approved by the Towson University Institutional Review Board (IRB #1810041426). Verbal and written informed consent were obtained for all participants and all study procedures adhered to those outlined in the Declaration of Helsinki. All participants provided consent to publish. This study was registed on ClinicalTrials.gov public website (NCT06163456).

## Funding

This study was funded by the Towson University Department of Kinesiology, the College of Health Professions Summer Undergraduate Research Institute, and the Towson University Faculty Development and Research Committee.

## Availability of data and materials

The datasets generated during and/or analyzed during the current study are available from the corresponding author on reasonable request.

## Authors' Contributions

**Rian Q. Landers-Ramos:** Conceptualization, Data curation, Formal analysis, Funding acquisition, Investigation, Methodology, Project administration, Resources, Software, Supervision, Validation, Visualization, Writing – original draft, Writing – review & editing. **Kathleen Dondero:** Data curation, Formal analysis, Supervision, Writing – review & editing. **Ian Imery:** Data curation, Writing – original draft. **Nicholas Reveille:** Data curation, Writing – original draft. **Hannah A. Zabriskie:** Formal analysis, Investigation, Methodology, Validation, Visualization, Writing – review & editing. **Devon A. Dobrosielski:** Formal analysis, Methodology, Validation, Visualization, Writing – original draft, Writing – review & editing.

## Conflict of interest

The authors declare that they have no known competing financial interests or personal relationships that could have appeared to influence the work reported in this paper.
